# Influence of Reciprocal Links in Social Networks

**DOI:** 10.1371/journal.pone.0103007

**Published:** 2014-07-29

**Authors:** Yu-Xiao Zhu, Xiao-Guang Zhang, Gui-Quan Sun, Ming Tang, Tao Zhou, Zi-Ke Zhang

**Affiliations:** 1 Web Sciences Center, University of Electronic Science and Technology of China, Chengdu, P. R. China; 2 Alibaba Research Center for Complexity Sciences, Hangzhou Normal University, Hangzhou, P. R. China; 3 Alibaba Research Institute, Hangzhou, P. R. China; 4 Complex Systems Research Center, Shanxi University, Taiyuan, Shanxi, China; 5 Department of Mathematics, North University of China, Taiyuan, Shanxi, China; University of Maribor, Slovenia

## Abstract

How does reciprocal links affect the function of real social network? Does reciprocal link and non-reciprocal link play the same role? Previous researches haven't displayed a clear picture to us until now according to the best of our knowledge. Motivated by this, in this paper, we empirically study the influence of reciprocal links in two representative real datasets, *Sina Weibo* and *Douban*. Our results demonstrate that the reciprocal links play a more important role than non-reciprocal ones in information diffusion process. In particular, not only coverage but also the speed of the information diffusion can be significantly enhanced by considering the reciprocal effect. We give some possible explanations from the perspectives of network connectivity and efficiency. This work may shed some light on the in-depth understanding and application of the reciprocal effect in directed online social networks.

## Introduction

Nowadays, the emergence of social networks and affiliated applications have triggered an increasing attention from various disciplines, ranging from studying the social interactions and spreading patterns in social sciences [1] to uncovering the underlying structure and dynamics in mathematics and physics [2]. Generally, social networks can be classified into two typical classes according to the edge properties: undirected and directed. Undirected social networks, such as *Flick* and *Okut*, do not allow two users to be connected unless the relation is mutually confirmed, hence, they are normally regarded as equivalent individuals in graph theory. Comparatively, directed social networks, such as *Twitter* and *Epinions*, contain both *unidirectional* and *bidirectional* links, which consequently build up a so-called *follower/followee* structure [3]. An online user is considered as a follower once he/she collects some other users as friends (followees), and puts close attention to them via automatically receiving their real-time information, as well as online activities [4]. A considerable fraction of those followees would also give positive feedback and add some of their followers with similar interests as online neighbors. Subsequently, such intermediate directed structure property, namely *reciprocity* [5], facilitates a great deal of attention from the scientific community. Nowak and Sigmund discussed that the indirect reciprocity would help in building reputation systems, judging morality and eventually promote the cooperation level [6] and benefit the evolution of natural selection [7] in both social environment [8] and supply networks [9]. Pereira *et al.* experimentally discussed that negative reciprocity, because of lower cost and less effort, was somehow more favored than the positive reciprocity [10]. Moreover, the power of reciprocity [11] does not only play a vital role in social economic systems [12] and human social organizations [13], [14], but also has been found wide applications in characterizing the property [15], [16], maintaining the structure [17], and uncovering the underlying function of directed social networks [18], [19]. Most recently, the network reciprocity has received outstanding attention in the realm of (co)evolutionary games [20]–[23] and the evolution of cooperation [24]–[26].

Typically, the simplest definition of reciprocity, 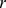, can be quantified as the ratio of the number of bidirectional links, 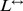, to the total number of links 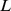 [27] (one bidirectional link is counted as two separate directed links),
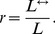
(1)


For the extreme cases, 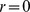 represents an absolute directed network where all links are unidirectional, and 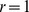 stands for a complete undirected network where all links are reciprocal. However, Garlaschelli and Loffredo [15] argued that Eq. (1) failed to precisely describe the full network information, For example, the network density and self-loops can significantly affect the final measurement of mutual connections. Alternatively, they proposed a new measure of reciprocity considering the ordering of different networks according to their actual degree of reciprocity, denoted as 
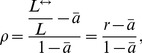
(2)where 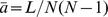 measures the ratio of observed links to all possible directed links (namely link density). Based on this improved measure, Zlatić *et al.* [16] reported that the reciprocity of Wikipedia could be very similar to other directed networks, but having a stronger reciprocity than the networks of associations and dictionary terms, and smaller than that of World Wide Web. Besides that, they found that such a measure is quite stable for different scales of Wikipedia networks, hence is very important for describing the structure and evolution of wiki-based networks. Boguñá *et al.* [28] found that reciprocal connections played a crucial role in constructing the giant connected component and possibly affecting the Web navigability. Futhermore, Serrano *et al.* [29] provided an in-depth study of the effect of reciprocal links on degree-degree correlations and clustering. They found that reciprocal links indeed organized the local subgraphs of the World Wide Web network by forming start-like structures, as well as cliques and communities, which contained highly interconnected pages. What's more, Gorka *et al.* [30] argued that the reciprocity was largely dependent on degree-degree correlation, which, consequently could partially reveal the underlying hierarchical structure of networks. Zlatić and Štefančić [31] discussed the influence of reciprocity on vertex degree distribution and degree correlations. They found that networks driven by reciprocal mechanisms are significantly different from static networks.

Recently, one revelent work tried to study the effect of reciprocal links in artificial networks [32]. While in this paper, we aim to provide a specific empirical study of the reciprocity influence on the function of real social networks. In particular, we apply a widely used epidemic spreading model [33], [34] to observe the effect of reciprocity on information spreading. Numerical results show that reciprocal links can noticeably enhance both the speed and coverage of information spreading compared with non-reciprocal links. In addition, we try to explain such phenomena by studying how reciprocal links affect the structure robustness as percolation catalysts in maintaining the global connectivity by investigating the avalanche of giant components, the network susceptibility and the network distance [35], [36].

## Data and Analysis

In this paper, we consider two representative directed social networks (datasets are free to download as Data S1): (i) *Sina Weibo*: the largest Chinese microblogging website, where a user (*follower*) can add others as his/her friends (*followee*) and automatically receive their posts and events. In addition, users can forward, comment or share their followees' news on their own post walls; The dataset was crawled through public APIs in March 2010. We start crawling with serval popular user, iteratively expanded to users who follow the crawled ones. (ii) *Douban*: the largest Chinese website for reviewing online movies, books, and music. Besides users' generally proactive contribution, *Douban* also provides services via its recommendation mechanism, which can suggest items of users' potential interests by mining their personalized preferences. Similar with *Sina Weibo*, users in *Douban* can also build follower-followee relationship with each other. The data analyzed was crawled through public APIs in Aug 2010, with starting crawl member lists of several hottest discussion groups, and iteratively expanded to users who follow or followed by the crawled ones [37], [38].

**Figure 1:**
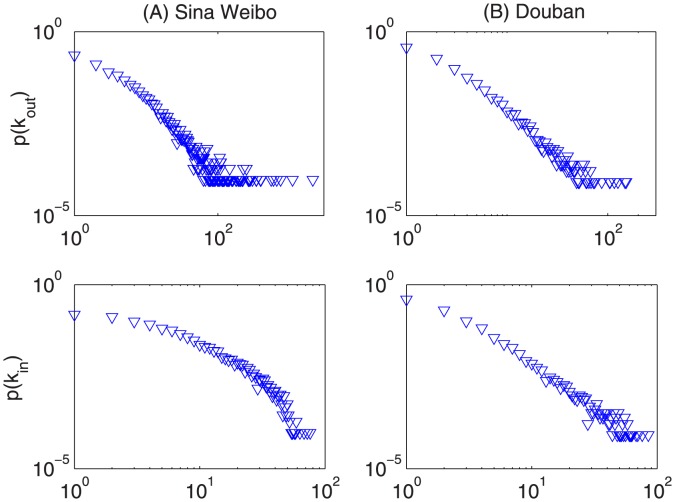
Out-degree(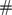
followers) and in-degree (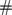
followees) distributions of the two observed data sets. Most users are ordinary beings who have relative small number of followers and keep only a small fraction of celebrities.

Consequently, such relationship can be represented by a directed network 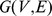, where 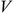 is the set of nodes and 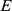 is the set of edges. Each node represents a user, and one link from user 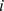 to user 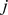 indicates 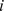 is followed by 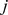, that is to say, 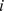 is the *followee* of 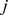, and 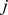 is one of 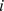
's *follower*. The two datasets both contain the *followship* information of almost hundred thousands of users. As previous work [39] demonstrated that 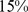 sampling is usually suitable to match the properties of the real graph, for the sake of balancing the integrity and computation complexity, we alternatively sample one manageable size to do analysis in this work. Table 1 summarizes the basic statistics of the largest connected component of sampled datasets. In addition, Fig. 1 shows the out-degree (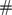 of followers) and in-degree (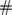 of followees) distributions, respectively. This common feature suggests that most users are ordinary beings who have relative small number of followers and keep only a small fraction of celebrities. Comparatively, the in-degree (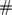 of followees) distribution of the two datasets does not exhibit the same phenomenon, which might suggest the different mechanisms driving the growth of two sites: information diffusing automatically in microblogging system of *Sina Weibo*, comparing with the information filtering by recommendation-related technique in *Douban*. Similar difference between passive and automatic patterns was also empirically reported in bipartite and hypergraph networks [40], [41]. In addition, we further investigate the average number of common follower and followees (see Table 2). Compared to non-reciprocal node pairs, reciprocal ones tend to have more common followers and followees, which is in accordance with previous work [19].

**Table 1:** Basic statistics of the two observed data sets.

Data sets	*N*	*L*	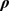	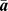
*Sina Weibo*	10,608	71,272	0.316	6.3 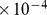
*Douban*	12,209	35,871	0.683	2.4 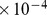


 and 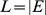 are the total number of nodes and links, respectively, 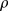 is the network reciprocity denoted by Eq. (2), and 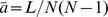 denotes the network sparsity.

**Table 2:** Comparisons of the average number of common followees (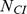) and followers (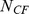) for reciprocal and non-reciprocal node pairs, respectively.

	Sina Weibo		Douban	
	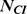	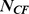	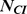	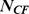
Reciprocal	**1.111**	**1.215**	**0.170**	**0.172**
Non-reciprocal	0.664	0.616	0.093	0.089

## Methods and Results

### Effect on Information Spreading

Information spreading [42] is one of the most important functions of social networks, where the information (messages, tweets, comments, etc.) can distribute at a remarkably fast speed through the whole online society via frequent interactions among users, although its structure is not designed on purpose for spreading news [43]. Up to now, there is a considerable number of theoretical models to study information diffusion on social networks [44]–[50]. Recently, one work showed that it's the fruitful interaction between hubs with many connections and average users with few friends, that make social networks are observed to spread information quickly. [51]. While in this paper, in order to understand the underlying mechanisms and possible factors that would result in the information outbreaks, we adopt one variant of the classic epidemic spreading model, *Susceptive-Infected* (SI) model [33], to evaluate the effect of reciprocal links in the two aforementioned social networks. We call this variant as *Directed Susceptive-Infected* (DSI) model. The diffusion process is described as following,

- Initially, user 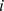 publishes an information item, *I*, in the corresponding social network. *I* could be about a piece of news, a photo, a comment, etc;
- All 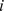
's followers will automatically receive *I* according to the *follower-followee* directed network structure. Then an arbitrary fraction of those followers might notice *I*, and forward it on their own homepages if they find it interesting. We consider this *forwarding willingness* as the *transmission probability*, denoted by 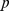;
- The above step will be repeated to the followers of 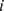
's followers, and eventually diffuses to the all achievable network nodes.

**Figure 2:**
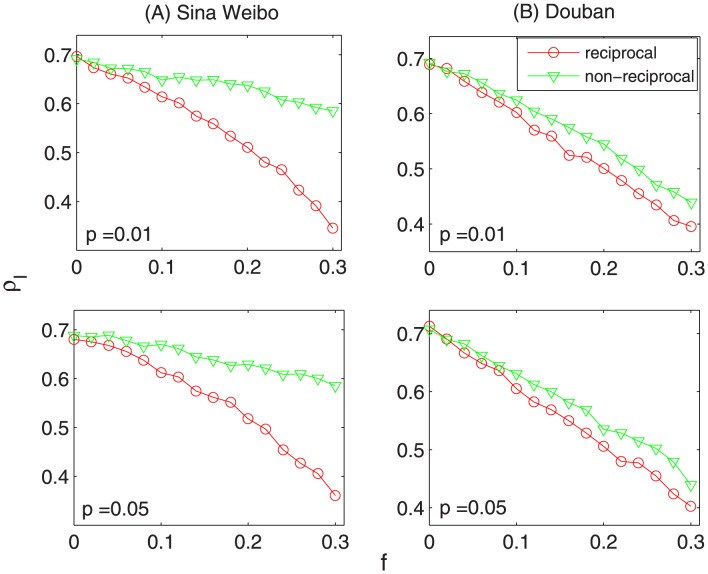
The fraction of influenced nodes as the function of the fraction of removed links 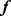. 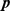 is transmission probability. In each subgraph, the red and green curves correspond to results of removing reciprocal and non-reciprocal links, respectively. Compared with removing non-reciprocal links, the fraction of influenced nodes 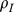 decays much faster when we remove the same amount of reciprocal links.

**Figure 3:**
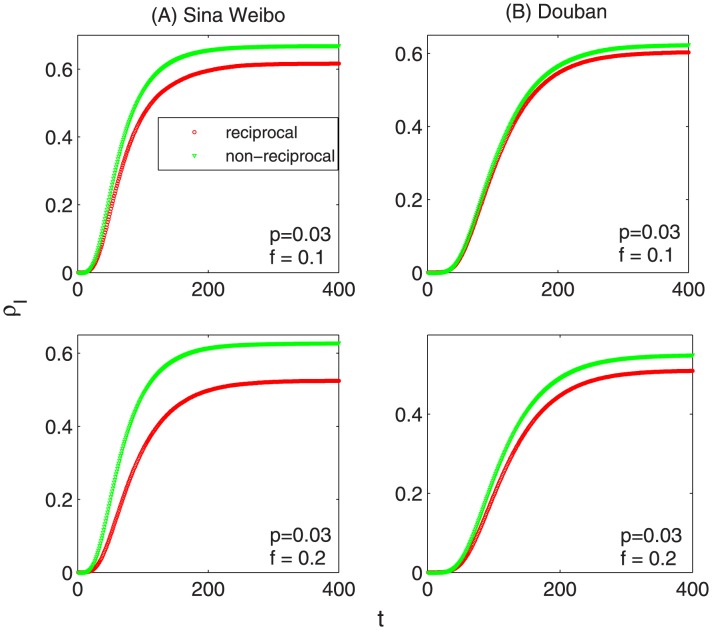
The fraction of influenced nodes as the function of observed time-step 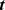, where 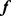 is the fraction of removed links and 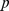 is transmission probability. The red and green curves correspond to results of removing reciprocal links and non-reciprocal links, respectively. Compared with removing non-reciprocal links, the diffusion speed is also affected much remarkably when removing reciprocal links.

Note that, the main difference between the *DSI* and classical *SI* model is that the link direction is taken into account. In the proposed *DSI* model, the information only can be transmitted from the followee to its own followers along with the direction of edges. Therefore, the final fraction of influenced nodes, 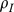, is determined by such a structure. In order to observe the effects of reciprocal links on information diffusion, we quantify the influence according to an edge percolation process [36], [52]–[54]. Obviously, if two reciprocal link (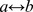) is more important than two separate non-reciprocal links (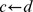 and 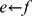), the information diffusion results will be affected significantly when we remove the same fraction of reciprocal and non-reciprocal links. That is to say, we seek to compare their differences via respectively removing an arbitrary amount of reciprocal links and the same number of non-reciprocal links (e.g., one reciprocal link is counted as two non-reciprocal links). Fig. 2 compares the information coverage of removing the two types of links. Compared with removing non-reciprocal links, 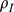 decays much faster when we remove the same amount of reciprocal links. Analogously, it also can be seen from Fig. 3 that the diffusion speed is affected much remarkably when removing reciprocal links. Therefore, it demonstrates that reciprocal links indeed play a more important role in the information diffusion process on directed social networks.

### Effect on Structural Robustness

As we all known, the famous weak tie theory shows that most people found job from acquaintance but not a friend [55]. While our results above indicate that reciprocal links (usually be strong ties) may play more important role in promoting the spreading of information than non-reciprocal links, which is inconsistent with weak tie theory. Why are reciprocal links more important for promoting the spreading of important? In conventional complex network theory, it is wildly agreed that the network function is largely influenced by its specific structure [56]. Therefore, to give solid and comprehensive understanding of the aforementioned results, we adopt the a dynamical removing process to measure the effects of reciprocal links on maintaining the structural robustness of networks [36]. For comparison, we apply three metrics to quantify the corresponding performance. (i) 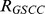: the size of the strongly connected giant component 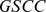 (the biggest community within which all nodes can be reached along with the link direction from any other node that is also in the same community). A sudden decline of 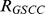 will be observed if the network disintegrates after deleting a certain fraction of edges; (ii) the network susceptibility (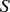): defined as
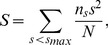
(3)where 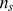 is the number of components with 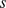 nodes, 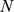 is the size of the network, and the sum runs over all the components except the largest one (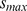). Note that, different with the definition in undirected networks, in Eq. (3), we only consider the strongly connected component in directed networks. Considering 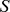 as the function of the fraction of removed edges 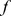. (iii) the average distance 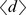, calculated by 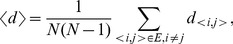
(4)where 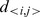 is the distance from node *i* to *j*. 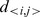 is set to 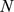 when there is no directed path from node *i* to *j*. Clearly, the smaller 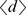 is, the better connectivity and more efficient the network will be.

**Figure 4:**
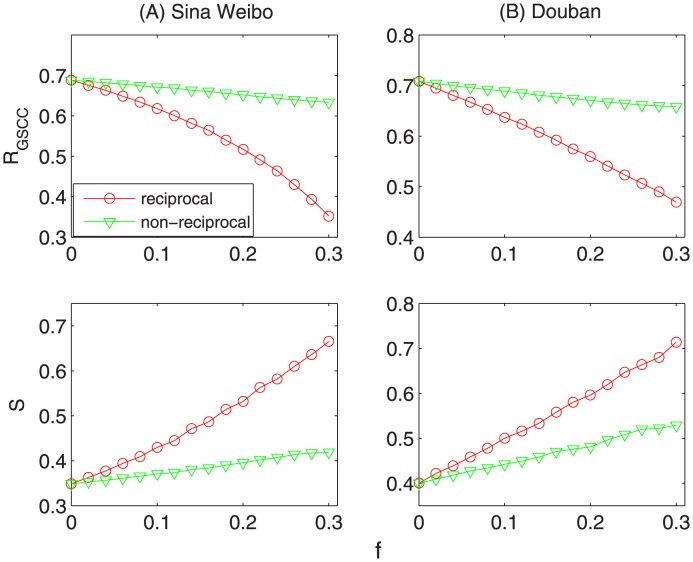
The fraction of giant component size (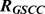) and the susceptibility (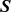) as the function of the fraction of removed links (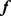) on the two observed datasets, (A) *Sina Weibo* and (B) *Douban*. In each subgraph, the red and green curves correspond to the results of reciprocal and non-reciprocal links, respectively. The size of strongly connected giant component (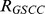) decreases more sharply when removing reciprocal links than deleting non-reciprocal ones. Accordingly, the network susceptibility (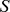) increase quicker when removing reciprocal links than that of deleting non-reciprocal links. That is to say, reciprocal links play a more important role in maintaining the connectivity of directed networks than non-reciprocal links.

**Figure 5:**
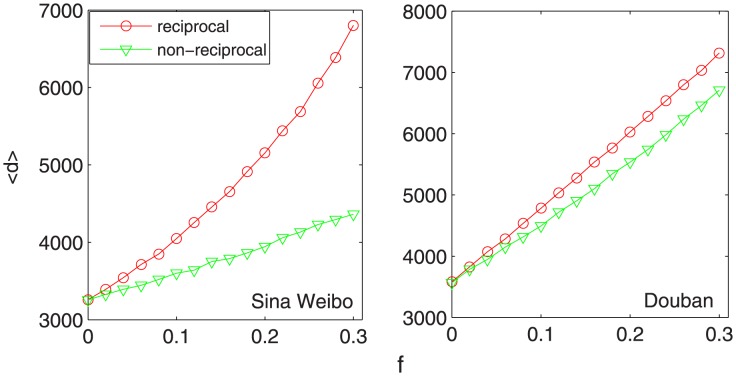
The average network distance (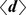) as the function of removed links (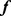) on the two observed datasets, (left panel) *Sina Weibo* and (right panel) *Douban*. The red and green curves correspond to the results of removing reciprocal and non-reciprocal links, respectively. The average network distance (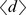) increases much faster when removing reciprocal links than deleting nonreciprocal ones. That is to say, reciprocal links also play a more important role in keeping the efficiency of directed networks than non-reciprocal links.

Fig. 4 and Fig. 5 show the corresponding results of the three examined matrices. In Fig. 4, it shows different dynamical patterns of removing reciprocal and nonreciprocal links, respectively. The size of strongly connected giant component (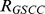) decreases more sharply when removing reciprocal links than deleting non-reciprocal ones. Accordingly, the network susceptibility (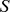) increase quicker when removing reciprocal links than that of deleting non-reciprocal links. In addition, Fig. 5 shows that the average network distance (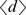) increases much faster when removing reciprocal links than deleting the nonreciprocal ones. In a word, different dynamical results indicate that reciprocal links play a more important role in both maintaining the connectivity and keeping the efficiency of directed networks than non-reciprocal links. It also strongly supports the results in the previous section that reciprocity can much promote the speed of information diffusion, as it takes a more significant responsibility for the robustness of directed networks. Furthermore, one interesting question is that what kind of nodes are more likely to have reciprocal links. Motivated by this, we calculate the correlation coefficient between the value of k-core (treat the network as undirected) and the proportion of reciprocal links. The correlation coefficient is 0.176 (0.23) for Sina Weibo (Douban), with 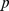-value 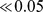, which indicates strong positive correlation between k-core number and proportion of reciprocal links. That's to say, nodes with higher probability to get reciprocal links tend to located at the core of the network.

## Conclusion and Discussion

In this paper, we have studied the influence of reciprocal links of directed networks from information spreading process. Experimental results on two representative directed social networks, *Sina Weibo* and *Douban*, show that reciprocal links indeed play a more important role than non-reciprocal ones. In particular, the results of information spreading show that reciprocity can significantly enhance both the spreading coverage and speed. We explain those phenomena by studying the effect of different type of links for network robustness. The two examined datasets show that the reciprocity is largely responsible for maintaining the connectivity and keeping the efficiency of directed networks, which suggests its significant impact in information spreading on networks.

The findings of this work may have a wide-range application in studying the role and influence of reciprocal links. Firstly, the topic of community detection has been well discussed [57], however, the progress on directed networks [58] is relatively slow. The main reason is that the modularity [59] of directed networks is rather difficult to be precisely defined. Secondly, most studies on epidemic spreading and information diffusion focus on studying the corresponding dynamics on undirected networks, the in-depth theoretical understanding of the underlying spreading mechanism on directed networks still remains to be solved. Finally, the area of information filtering confronts a huge challenge as more and more directed social services are provided in the information era. The present work just provides a start point to see the preliminary effects of reciprocal links, a more comprehensive and in-depth understanding of reciprocity still need further efforts to discover.

The findings of this work may have a wide-range application in studying the role and influence of reciprocal links. Firstly, the topic of community detection has been well discussed [57], however, the progress on directed networks [58] is relatively slow. The main reason is that the modularity [59] of directed networks is rather difficult to be precisely defined. Secondly, most studies on epidemic spreading and information diffusion focus on studying the corresponding dynamics on undirected networks, the in-depth theoretical understanding of the underlying spreading mechanism on directed networks still remains to be solved. Finally, the area of information filtering confronts a huge challenge as more and more directed social services are provided in the information era. The present work just provides a start point to see the preliminary effects of reciprocal links, a more comprehensive and in-depth understanding of reciprocity still need further efforts to discover.

## Supporting Information

Data S1 **Data sets.**
(ZIP)
